# In the spotlight: the role of TGFβ signalling in haematopoietic stem and progenitor cell emergence

**DOI:** 10.1042/BST20210363

**Published:** 2022-03-14

**Authors:** Roshana Thambyrajah, Rui Monteiro

**Affiliations:** 1 Stem Cell and Cancer Group, IMIM, Barcelona, Spain; 2 Institute of Cancer and Genomic Sciences, College of Medical and Dental Sciences, University of Birmingham, Birmingham, U.K.; 3 Birmingham Centre of Genome Biology, University of Birmingham, Birmingham, U.K.; 4 Cancer Research UK Birmingham Centre, Birmingham, U.K.

**Keywords:** BMP, endothelial to haematopoietic transition, haemogenic endothelium, mouse models, TGFβ, zebrafish

## Abstract

Haematopoietic stem and progenitor cells (HSPCs) sustain haematopoiesis by generating precise numbers of mature blood cells throughout the lifetime of an individual. In vertebrates, HSPCs arise during embryonic development from a specialised endothelial cell population, the haemogenic endothelium (HE). Signalling by the Transforming Growth Factor β (TGFβ) pathway is key to regulate haematopoiesis in the adult bone marrow, but evidence for a role in the formation of HSPCs has only recently started to emerge. In this review, we examine recent work in various model systems that demonstrate a key role for TGFβ signalling in HSPC emergence from the HE. The current evidence underpins two seemingly contradictory views of TGFβ function: as a negative regulator of HSPCs by limiting haematopoietic output from HE, and as a positive regulator, by programming the HE towards the haematopoietic fate. Understanding how to modulate the requirement for TGFβ signalling in HSC emergence may have critical implications for the generation of these cells *in vitro* for therapeutic use.

## Introduction

TGFβ is a well-known regulator of adult haematopoietic stem cell (HSC) lineage determination, self-renewal and differentiation in development and in disease [1–4]. Its functions in adult haematopoiesis have been discussed in many excellent reviews [4–6] and will not be covered here. Here we will focus on the emerging role of TGFβ signalling in the formation of nascent HSPCs during embryonic development, the current views regarding TGFβ signal transduction and transcriptional responses in this context, and future research that can contribute to resolve the existing concerns and contradictions.

### The fundamentals of TGFβ signalling — canonical and non-canonical signalling

**Figure 1.**
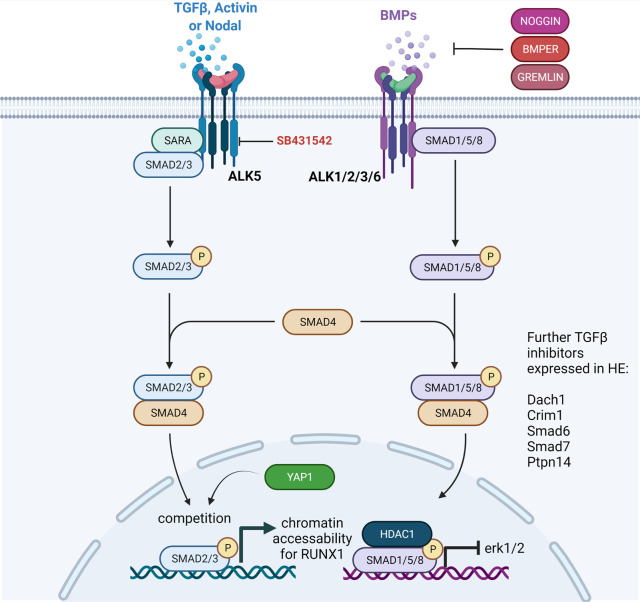
Schematic representation of TGFβ signalling. The TGFβ ligands bind and activate type II receptors that phosphorylate type I receptors (ALK1–7). The ALKs in turn phosphorylate intracellular mediators (ALKs). Alk1/4/5 phosphorylate SMAD2/3, whereas Alk1/2/3/6 can phosphorylate SMAD1/4/8 in endothelial cells. The compound SB431542 can inhibit ALK5. The phosphorylated SMAD form a complex with the common SMAD4 to regulate gene transcription. Alternatively, SMAD2/3 can form a complex with YAP1 instead of SMAD4 to act as a chromatin pioneering factor. SMAD1/5/8 recruits HDAC proteins to the promoter erk1/2 gene. NOGGIN, BMPER and GREMLIN1a expression show an inverse correlation to pSMAD1/5/8 staining.

TGFβ proteins are part of a large family of secreted growth factors with pleiotropic activities in diverse tissues ranging from proliferation, cell death and differentiation to lineage determination, organ morphogenesis and tissue homeostasis [5,7,8]. The family comprises over 30 members, including TGFβ1–3, Activins, Bone Morphogenetic Proteins (BMPs) and Growth and Differentiation Factors (GDFs) [9,10]. The fundamental components of this pathway have been elucidated over the years by many research groups and have been excellently reviewed by many colleagues. Briefly, in canonical signalling, TGFβ secreted factors bind to heteromeric tyrosine kinase type I and type II receptor complexes at the cell surface. Upon ligand binding, type II receptors phosphorylate type I receptors, which in turn phosphorylate intracellular mediators called Receptor-regulated Smad proteins (R-Smads). Subsequently, R-Smads then form a complex with the common Smad, Smad4, and translocate to the nucleus and bind to specific promoters and enhancers to regulate gene expression [9]. There are seven type I receptors (ALK1–ALK7) and five type II receptors (TGFβRII, BMPRII, ActRII, ActRIIB and AMHRII) and different TGFβ ligands bind different combinations of type I and type II receptors [11]. TGFβ, Nodal and Activin generally induce phosphorylation of Smad2 and Smad3 via ALK4 or ALK5, whereas BMPs phosphorylate Smad1, Smad5 or Smad8 through ALK2, ALK3 or ALK6 [7] (Figure 1). In endothelial cells, however, TGFβ can bind receptor complexes containing either ALK5 (TβRI) or ALK1 (ACVRL1) activating Smad2 and Smad3 via ALK5, but also Smad1 and Smad5 via ALK1 [12–14]. In addition to the canonical Smad-mediated pathways, there are several non-smad mediators of TGFβ signalling, including the c-Jun amino terminal kinase (JNK), p38 MAPK, Akt and others, collectively known as non-canonical signalling mediators and are reviewed elsewhere [6,15].

### HSPCs arise from haemogenic endothelium during embryonic development

HSPCs sustain the blood system throughout life. Although they reside in the bone marrow of the adult, their origin can be traced to a region where the aorta, gonads and the mesonephros (AGM) meet in vertebrate embryos. Elegant time lapse imaging, transplantation assays, immuno-histochemistry for HSPC-associated markers, gene expression, and more recently single cell transcriptomics have convincingly demonstrated the existence of specialised endothelial cells with haematopoietic stem and progenitor cell (HSPC) potential, termed hemogenic endothelium (HE), that undergo a endothelial to haematopoietic transition (EHT) from the lining of the dorsal aorta and thereby progressively lose their endothelial identity and become haematopoietic [16–22]. These cells form intra-aortic haematopoietic clusters (IAHC) that appear associated with the ventral wall of the dorsal aorta in murine embryos starting between the embryonic days 10.25–12 (E10.25–E12). The emerging HSPCs then migrate to the foetal liver for maturation and expansion [23–27]. The sites of HSPC emergence and their migration between haematopoietic niches are very well conserved in vertebrates [28]. Based on transplantation assays performed at different time points within the window of HSPC emergence, early pre-HSC can readily contribute to the blood system of neonates, but not adult [29]. This potency is only evident in HSCs that are older than E11.5 and even then, only a very small fraction of these cells are functional HSCs [30–33]. Therefore, although there is consensus regarding the site of HSPC emergence, it is unclear whether HSPCs and HSC share the same HE precursors or if in fact the HE itself is heterogeneous, and which of the molecular pathways are unique to HSC emergence or shared amongst all HSPCs. In this review, we collectively discuss the role of TGFβ superfamily signalling in the emergence of all HSPC within the dorsal aorta, since there are no established makers to distinguish between haematopoietic progenitors and HSCs. In addition, EHT is a continuous process, meaning that the cells in the AGM are at different stages of generating HSPC. Adding to the complexity, EHT occurs at a developmental time when angiogenic processes are still taking place and vascular identity is being established. Therefore, it is highly likely that both these processes share common signalling pathways. In fact, despite some evidence suggesting that these two cell types may be independent from each other [34–36], it is now widely accepted that arterial specification of the aorta is a pre-requisite for HE specification and subsequent EHT of HSPCs [37–40].

### Expression of TGFβ signalling pathway components during the formation of HSPCs

The expression of TGFβ superfamily members has been assessed in several *in vivo* and *in vitro* model systems for haematopoiesis. The TGFβ receptors TGFβRII, ALK1 and ALK5 are present in the endothelial cells and HE derived from Embryonic stem cells (ES) *in vitro* [41], zebrafish [42,43], mouse and chick embryos [41,44]. Endoglin, a type III co-receptor for both TGFβ and BMP9/BMP10 [13,45–47], is also expressed in endothelium [12,41,44]. Of the 3 TGFβ ligands, TGFβ1 (including *tgfβ1a* and *tgfβ1b* in zebrafish) is the most prominent one with high levels of expression in the mouse, chick, zebrafish and ESC-derived endothelium and HE [41,42,44,48]. Only very low levels of *tgfβ2* have been detected in mouse endothelium, and *tgfβ3* is hardly detectable in either the mouse or the chick endothelium [41,44]. However, both ligands are highly abundant in the sub-aortic mesenchyme and the notochord in chick [44] and in zebrafish [42]. In zebrafish, *tgfβ3* was additionally detected in the endocardium, somites, floor plate and notochord [42].

### The role of TGFβ signalling in the formation of HSPCs

Several labs have studied the involvement of TGFβ signalling in HE specification, EHT and IAHC formation and have come to seemingly contradictory conclusions. The signalling triggered by TGFβ ligands has been studied for its functional requirement during EHT in different models. Several studies have identified the expression of SMAD2/3 in the dorsal aorta and HE and provided evidence for activated SMAD2/3 (pSMAD2/3) in mouse HE/IAHC [49]. Both in mouse and chick embryos only a very discrete number of cells show pSMAD2/3 staining [44,49]. It is plausible that the snapshots provided by the IHC experiments do not represent the overall level of active SMAD2/3 induced by TGFβ ligands in HE since the trigger can by limited to a small window of time during HE specification and EHT.

Functional studies with morpholino mediated knock down of TgfβRII, TGFβ1a or TGFβ1b or using genetic *tgfb1b* mutants during zebrafish haematopoiesis leads to impaired specification of the HE [42,48]. Knockdown of TGFβ3 led to impaired EHT and decreased haematopoietic output from HSPCs [42]. In contrast, pharmacological inhibition of TGFβ signalling with the compound SB431542 increased haematopoietic output in a mouse embryonic stem cell (mESC) haematopoietic differentiation model [41]. This might seem contradictory at first, but a detailed study of the effects of SB431542 on pSMAD2/3 indicated that in cultures exceeding 24 h, the use of the inhibitor increases SMAD2/3 phosphorylation [49]. Indeed, overexpression of constitutively active SMAD2/3 resulted in the same phenotype as adding the inhibitor. It is unclear how the compound can increase the levels of pSMAD2/3 [50]. It's tempting to hypothesise that blocking the ALK5 receptor leads to over-activation of ALK4/7 that in turn phosphorylate SMAD2/3 as a fine balance is maintained between inputs from different ALKs in a cell [51]. A recent study using haematopoietic differentiation of human ES cells did not report an increased haematopoietic output when using the inhibitor in >24 h culture [52]. However, the authors used a lower concentration of the inhibitor compared with others and neither of these studies added the inhibitor at equivalent stages of *in vitro* differentiation (i.e. Bruveris et al. from the PDGFRα+ (mesodermal) stage, Thambyrajah et al. from the Flk1^+^ stage (mesodermal/haemangioblast) and finally Vargel et al. from the EHT stage (CD144^+^/CD41^+^) [41,49,52]. Beyond the issues with the mode of action of the inhibitor, these differences make direct comparisons to TGFβ function *in vivo* difficult. Vargel et al. also added the ligand TGFβ2 to Flk1^+^ cells sorted from embryoid body cultures which resulted in a decrease in VE-cad^−^/CD41^+^ haematopoietic progenitor cells (HPCs). They therefore postulated that TGFβ signalling blocked the formation of blood cells and thus negatively regulates haematopoiesis [41]. In contrast, using TGFβ1 in a similar ESC differentiation model led to increased number of CD41^+^/CD117^+^ HSPCs [48]. Here it is important to note that TGFβ1 is the main ligand in angiogenesis and haematopoiesis [42,53–55]; although TGFβ2 is a key inducer of endothelial to mesenchymal transition (EndoMT) in the heart [56], it is not expressed in the aortic endothelium or HE [41,42,44]. In addition, mouse mutants for *Tgfb2* don't show any obvious angiogenic or haematopoietic defects [57]. TGFβ2 knockdown had no effect on HE specification in zebrafish either [42].

Further experimental evidence for a crucial involvement of SMAD2/3 in EHT was reported in two very recent studies. Here, they identify SMAD2/3 interactions with the chromatin as vital for transcription factors to access their target sites during EHT. In fact, in both studies, SMAD2/3 is discovered as a pioneering factor for opening the chromatin for cell fate change, including for the key haematopoietic transcription factor Runx1 [58,59]. Accordingly, HE and IAHC that are positive for pSMAD2/3 have been reported in the mouse AGM [49]. A more complex function for SMAD2/3 in EHT can be anticipated from studies on EndoMT. In EndoMT, the Hippo pathway member YAP1 can compete with Smad4 for complex formation with Smad3 during cardiac cushion formation [60]. The study finds that YAP1/Smad3 complexes have a stronger DNA binding affinity and drive stable transcription of downstream targets [60]. It´s noteworthy that YAP1 induced by shear stress is required for HSPC/HSC maintenance in the zebrafish AGM [61]. It will be interesting to examine if YAP1 interacts with Smad3 to induce and maintain HSPC/HSC fate during AGM haematopoiesis. Finally, several TGFβ regulatory proteins such as Smad6, Smad7, Bmper, Dach1, Crim1 and Ptpn14, are expressed in ES Cell-derived HE and are targets of the histone deacetylases Hdac1 and/or Hdac2 [49]. Interestingly, ChIP assays demonstrated that Runx1 binds to the Smad6 -57 enhancer and drives its expression in aortic endothelial cells [62]. Here, we hypothesise that Smad6 expression may be silenced in EHT-primed HE through histone deacetylases Hdac1/Hdac2. Histone silencing complexes can be recruited by transcription factors, including Gfi1 and its homologue Gfi1b [63–65]. Therefore, Runx1-expressing cells that start to express Gfi1/1B would recruit histone deacetylases (or other histone modifiers) to repress the negative regulators of TGFβ and permit TGFβ activity. Since epigenetic modifiers can be recruited by various transcription factors, it is possible that several HE and later HSPC-specific transcription factors fine tune the overall output of TGFβ signalling in each cell, and that the output can be varied depending on the cell type.

### Evidence for non-canonical TGFβ signalling in HSPC emergence

**Figure 2.**
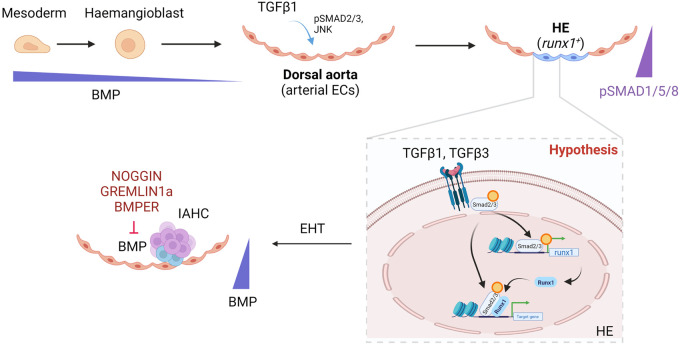
Schematic representation of the stepwise development from mesodermal cells to HSPC/HSCs in the vertebrate embryo and the requirement of TGFβ signalling in these transitions based on current studies. Decreasing BMP4 levels are needed from the mesodermal to a HE stage, and as a gradually decreasing gradient from the sub-aortic mesenchyme toward the ventral wall of the dorsal aorta. Within the ventral wall of the dorsal aorta, HE/HSPC/HSCs cells are activated by TGFβ1/3 and show presence of pSMAD2/3 and low pSMAD1/5/8. BMP4 is antagonised by BMPER, GREMLIN1a and NOGGIN. In our hypothesis, based on the published data, we postulate that SMAD2/3 is needed to open the chromatin for Runx1 to drive EHT, in a process similar to that seen in EndoMT. Please note that the scheme does not include other known regulators of Runx1 expression such as Notch or VegfA signalling.

Zhang and colleagues showed that Tgfβ1b regulated HSPC emergence by promoting gluconeogenesis, an effect mediated via the non-canonical JNK/c-jun pathway rather than canonical Smad2 phosphorylation [48]. They demonstrated that the expression of zebrafish JNK orthologues *tak1*, *mapk8a*, *mapk9* and *mapk10* was decreased in *tgfb1b* mutant endothelial cells, suggesting a role for JNK downstream of TGFβ signalling. Accordingly, chemical inhibition of JNK signalling phenocopied the loss of HSPCs found in *tgfb1b* mutants. They further identified that *c-jun* expression was decreased in the mutants [48]. Loss of *tgfb1b* or *c-jun* led to decreased *g6pc3*, an enzyme in the gluconeogenesis pathway that was specifically enriched in HSPCs. Addition of 1% glucose or re-expression of *g6pc3* rescued expression of HSPC-specific markers *runx1* and *cmyb* [48]. They proposed that a Tgfβ1b/JNK/c-jun/g6pc3 axis is required to maintain sufficient levels of gluconeogenesis to enable HSPC emergence from HE. Together with our previous study in zebrafish [42], these experiments indicate that TGFβ signalling is an inducer of HE and is critical for the formation of HSPCs (Figure 2).

### The role of BMP signalling in the formation of HSCs

More research has been conducted to understand the role of BMP-induced signalling through ALK2, ALK3 or ALK6 in the generation of HSPCs. While BMP4 expression surrounds the mesenchyme around the dorsal aorta, BMP activity traced with a Smad1/5-responsive reporter mouse line is detected in cells of the dorsal aorta and HSPCs, with all HSC activity residing in the BMP activated cell AGM population [66]. This stark distinction is lost in the later stages of HSCs maturation where HSC activity resides in both the BMP-activated and non-activated cell fraction, although BMP activated HSCs are more enriched for myeloid biased HSCs [66]. BMP is required to establish the haematopoietic programme in definitive haemangioblasts (the precursors of the dorsal aorta and HE in *Xenopus* embryos), but not thereafter [67]. These observations tie in with reports that identified expression of the BMP antagonists, Noggin2, gremlin1a and Bmper, at an increasing gradient towards the aorta in the mesenchyme below. At least *noggin2* and *gremlin1a* are induced by FGF signalling from the somites during zebrafish haematopoiesis [68]. Overexpression of noggin from pre-mesoderm stages in *Xenopus* embryos abrogates HE specification and blood development from the dorsal aorta but has no effect on haematopoiesis if induced after the event [67]. This indicates a time-limited requirement for BMP signalling during HSPC emergence (Figure 2). Similarly, the expression of *Bmper* increases just after the specification of HE, between E9.5 and E11.5 in a ventrally polarised manner in the AGM. When comparing the localisation of BMPER and BMP-activated cells (indicated by nuclear pSMAD1/5/8 immunostaining) within the AGM region, they showed a negatively correlated distribution, further indicating that Bmper restricts BMP4 activation in emerging IAHC [69]. Nevertheless, *Bmper* mRNA can be occasionally detected in some intra-aortic cells, including the early cells in the IAHC. Within the IAHC, Noggin expression also shows an inverse correlation with nuclear pSMAD1/5/8 immunostaining [69]. Notably, the TGFβ receptor(s) driving this Smad 1/5/8 phosphorylation in the AGM was not identified in these studies. It remains a possibility that next to BMP4, TGFβ ligands contribute this Smad activation, since they can phosphorylate Smad1 and Smad5 via ALK1 [12–14]. One of the experimentally validated downstream targets of active BMP4 signalling during HE specification and EHT is Erk1/2 signalling [70], a tyrosine kinase receptor activated pathway that controls proliferation and survival [71]. The balance between the Erk1/2/MAPK and the PI3K/AKT pathway determine the arterial versus venous specification [72] in angiogenesis. Zhang et al. [70] revealed that pSMAD1/5 accumulate at the promoter regions of erk1 and erk2 to repress gene expression through recruitment of epigenetic modifiers, including Hdac1. Here again, it is interesting that Erk activity is needed at early stages of HE/EHT. Altogether, these findings suggest an early need for BMP4 signalling prior to HE specification that becomes dispensable thereafter. In fact, pharmacological inhibition of Erk after this stage has the opposite effect, leading to the hypothesis that there is switch from a positive to negative requirement for Erk signalling during embryonic haematopoietic development [70].

## Concluding remarks and future directions

This review highlights the pivotal roles played by the TGFβ family of signalling molecules during HE specification, EHT and HSPC/HSC emergence that have been characterised in the recent years. The evidence that has emerged from different model systems conclusively support a dynamic and carefully timed requirement for TGFβ signalling. Most reports clearly support a critical input from pSMAD2/3 and pSMAD1/5/8 for HSPC emergence and there is mounting evidence that non-canonical signalling may also play an important part in this process [48] (Figure 2). Remarkably, a novel interaction between Runx1 and SMAD2/3 has recently been discovered that highlights a key role for TGFβ signalling in promoting EHT by mediating chromatin accessibility of Runx1 target genes [58]. Transcription factor footprinting of haemogenic (E9.5) and non-haemogenic (E13.5) endothelium revealed higher enrichment of Smad2/3 binding motif next to the Runx1 motif at E9.5 [58]. Activation of Runx1 together with TGFβ3 was sufficient to induce HE activity in E13.5 endothelial cells, indicating that these cells progressively lose their plasticity as they mature. Accordingly, adult endothelium stayed refractory to transient induction of combined Runx1 and TGFβ3 expression [58], indicating that other factors might play a role in maintaining that haemogenic plasticity observed at earlier developmental stages. Because recent studies demonstrated the existence of combinatorial SMAD2/3 and Smad1/5-mediated signalling during EMT [14], we speculate that TGFβ-mediated Smad1/5 signalling may also contribute to HE specification and/or EHT. In this regard, *in vitro* differentiation of embryoid bodies from mouse ES cells towards blood showed increased numbers of CD45^+^ ALK1^+/−^ cells compared with wild-type [44], suggesting a role for ALK1 in balancing the arterial versus haemogenic cell fate. This observation remains to be confirmed *in vivo*.

Overall, an abundant number of positive and negative TGFβ family regulators are expressed in the AGM region and therefore, further investigation is required to explain how the entire network is spatially, temporally and functionally coordinated during the stepwise development of HE to EHT and finally to HSPCs/HSCs. Linking data from the growing number of single cell transcriptional profiling studies in HE/HSPCs (e.g. [73–77]) with further studies on the exact composition of SMAD complexes will help address these questions. Chromatin binding data for the individual Smad proteins in HE or HSPC/HSCs would be desirable, but will remain a challenge to perform in low cell number samples until good antibodies and new alternatives to conventional ChIP such as Cut&Tag [78] are standardised. Moreover, Smad proteins only bind to DNA transiently and need transcription factors to stabilise these interactions that additionally increases the difficulty in identifying bona fide binding targets [60,79]. Finally, there is still a need to develop specific compounds to activate or inhibit specific TGFβ receptors and ligands since TGFβ signalling is aberrantly expressed in many diseases [80]. The first studies and clinical trial using the novel TGFβ1/3 inhibitor AVID200 in patients with advanced solid tumours show encouraging results [81,82]. The use of such compounds will help to broaden our understanding of TGFβ signalling in the development of HSPC from HE. In summary, we have identified and discussed mounting evidence for a central role for TGFβ signalling at the earliest stages of HSPC emergence. Which specific Smad complexes direct certain stages of HE specification, EHT and HSPC differentiation and which downstream targets are involved is yet to be fully unravelled.

## Perspectives

- Signalling by the Transforming Growth Factor β family is crucial for the establishment of haematopoietic stem and progenitor cells (HSPCs) from haemogenic endothelium in the developing embryo.
- While there are controversial observations suggesting opposing roles for TGFβ in the formation of HSPCs, the consensus is emerging that TGFβ signalling is required to programme the embryonic arterial endothelium towards the haematopoietic fate.
- A better understanding of the activities of TGFβ signalling that enable HSPC emergence will help deliver on the promise of generating these cells *in vitro* for personalised medicine applications. New cutting-edge technologies such as single cell transcriptomics and epigenomics and better techniques to interrogate transcription factor binding in very low cell numbers will provide a solid platform to achieve that goal.

## Open Access

Open access for this article was enabled by the participation of University of Birmingham in an all-inclusive *Read & Publish* agreement with Portland Press and the Biochemical Society under a transformative agreement with JISC.

## Abbreviations

ActRII: Activin receptor type II
AGM: Aorta-Gonad-Mesonephros
AKT: Ak strain transforming
ALK: Activin-like kinase
AMHRII: Anti-Mullerian Hormone receptor type II
BMP: Bone Morphogenetic Protein
BMPRII: BMP receptor type II
ChIP: Chromatin Immunoprecipitation
EHT: Endothelial to Haematopoietic Transition
EMT: Epithelial to Mesenchymal Transition
EndoMT: Endothelial to Mesenchymal Transition (EndoMT)
ERK: Extracellular Signal-Regulated Kinase.
GDF: Growth and Differentiation Factor
HE: Haemogenic endothelium
HSC: Haematopoietic Stem Cell
HSPC: Haematopoietic Stem and Progenitor Cell
IAHC: Intra-aortic Haematopoietic Cluster
JNK: c-Jun amino terminal kinase
MAPK: Mitogen-activated protein kinase
mESC: mouse embryonic stem cell
PI3K: Phosphatidil inositol 3’-kinase
pSMAD1/5: phosphorylated SMAD1/5
pSMAD2/3: phosphorylated SMAD2/3
TGFβ: Transforming Growth Factor β
TGFBRII: TGFβ receptor type II
